# Association between the fatty liver index and the risk of severe complications in COVID-19 patients: a nationwide retrospective cohort study

**DOI:** 10.1186/s12879-022-07370-x

**Published:** 2022-04-17

**Authors:** Yoonkyung Chang, Jimin Jeon, Tae-Jin Song, Jinkwon Kim

**Affiliations:** 1 Department of Neurology, Mokdong Hospital, Ewha Woman’s University College of Medicine, Seoul, 07985 Republic of Korea; 2 Department of Neurology, Yongin Severance Hospital, Yonsei University College of Medicine, 363, Dongbaekjukjeon-daero, Giheung-gu, Yongin-si, 16995 Republic of Korea; 3 Department of Neurology, Seoul Hospital, Ewha Woman’s University College of Medicine, 260, Gonghang-daero, Gangseo-gu, Seoul, 07804 Republic of Korea

**Keywords:** COVID-19, Fatty liver index, Non-alcoholic fatty liver, Mechanical ventilation, Prognosis

## Abstract

**Background:**

Research on the association of non-alcoholic fatty liver disease (NAFLD) with prognosis in COVID-19 has been limited. We investigated the association between the fatty liver index (FLI), a non-invasive and simple marker of NAFLD, and the severe complications of COVID-19 patients in South Korea.

**Methods:**

We included 3122 COVID-19-positive patients from the nationwide COVID-19 cohort dataset in South Korea between January and June 2020. The FLI was calculated using triglyceride, body mass index, glutamyl transpeptidase, and waist circumference, which were obtained from the national health screening program data. Severe complications related to COVID-19 were defined as the composite of mechanical ventilation, intensive care unit treatment, high-oxygen flow therapy, and death within 2 months after a COVID-19 infection. We performed a multivariate logistic regression analysis for the development of severe complications in COVID-19 patients.

**Results:**

The mean ± standard deviation of FLI were 25.01 ± 22.64. Severe complications from COVID-19 occurred in 223 (7.14%) patients, including mechanical ventilation in 82 (2.63%) patients, ICU admission in 126 (4.04%), high-flow oxygen therapy in 75 (2.40%), and death in 94 (3.01%) patients, respectively. The multivariate analysis indicated that the highest tertile (T3) of FLI was positively associated with severe complications from COVID-19 (adjusted odds ratio (OR): 1.77, 95% confidence interval (CI) (1.11–2.82), P = 0.017) compared with the lowest tertile (T1).

**Conclusions:**

Our study demonstrated that FLI, which represents NAFLD, was positively associated with an increased risk of severe complications from COVID-19. FLI might be used as a prognostic marker for the severity of COVID-19.

## Background

The coronavirus disease of 2019 (COVID-19) caused by severe acute respiratory syndrome coronavirus 2 (SARS-CoV-2) became the greatest health and economic threat in the twenty-first century [1, 2]. The prognosis of most patients infected with COVID-19 is good, though a significant proportion of patients experience critical complications. COVID-19 patients with complications may require mechanical ventilation or hospitalization in intensive care units, and some can become fatal. As of July 30, 2021, over 196 million global cases of COVID-19 infection had occurred, leading to more than 4 million COVID-19-related deaths [3]. Previously, there have been several studies investigating those at higher risk of a COVID-19 infection alongside the patients at risk of severe complications from COVID-19. Elderly age, hypertension, diabetes mellitus, insulin resistance, cancer, lung disease, chronic kidney disease, and cardiovascular disease are known factors related to severe complications from COVID-19 [4–6]. Furthermore, the risk of severe COVID-19 increases in conjunction with the number of underlying health conditions [7].

Non-alcoholic fatty liver disease (NAFLD) is the most prevalent chronic liver disease worldwide and a metabolic condition in which fat accumulates in the liver related to insulin resistance [8]. Moreover, it is not promoted by excessive alcohol consumption [8]. NAFLD was previously considered the intrahepatic phenotype of metabolic syndrome. Recently, however, NAFLD has been presented as an independent risk factor for several diseases. Indeed, NAFLD is closely associated with cardiovascular diseases as well as hypertension, diabetes mellitus, and chronic kidney disease [9–12], which are closely related to a poor prognosis after COVID-19 infection. Meanwhile, the fatty liver index (FLI) is a simple algorithm based on body mass index (BMI), waist circumference, triglycerides, and gamma-glutamyl transferase (GGT), which has been proposed as a useful surrogate marker and predictor of NAFLD [13]. Previous studies have shown that the presence, severity, and progression of NAFLD are significantly associated with FLI [14, 15].

Thus, we hypothesized that FLI levels could predict the severe complications occurring from COVID-19. Therefore, this study aimed to investigate the association between FLI levels and severe complications in patients infected with COVID-19 in a nationwide retrospective cohort.

## Methods

### Study design and data

A retrospective cohort study was performed with a nationwide population-based COVID-19 dataset in South Korea. During the COVID-19 pandemic, the South Korean government gathered health insurance data of people who underwent SARS-CoV-2 PCR testing between 2020.1 and 2020.6 for academic purposes (https://hira-covid19.net/). This COVID-19 dataset included people living in South Korea who had undergone a real-time RT PCR assay of either a nasal or pharyngeal swab. The real-time PT PCR assay kit followed the WHO guidelines and was validated by the Korean Centers for Disease Control and Prevention [16]. Since NHIS is a single-payer public health care system in South Korea, the COVID-19 dataset contains data on demographics, national health screening examinations, hospital visits, diagnoses, medications, procedures, and death [17].

To calculate the FLI index, data collected from the South Korean national health screening program between January 2015 and December 2019 was used, which was directly linked to the COVID-19 database [18–21]. It should be noted that if the health screening examination was performed twice or more during this period then the most recent result was used in this study. The NHIS provides a biannual complimentary nationwide health checkup program to all Koreans aged ≥ 40 years [18]. The health screening program dataset includes information on demographics, physical examinations, blood tests, lifestyle, and health history questionnaires [21].

### Ethics statement

This study was approved by the Institutional Review Board of our institute (Seoul Hospital Ewha Woman’s University College of Medicine 2020-10-021). Furthermore, the requirement for informed consent was waived due to the retrospective analysis, which was performed on a fully anonymized dataset.

### Participants

**Fig. 1:**
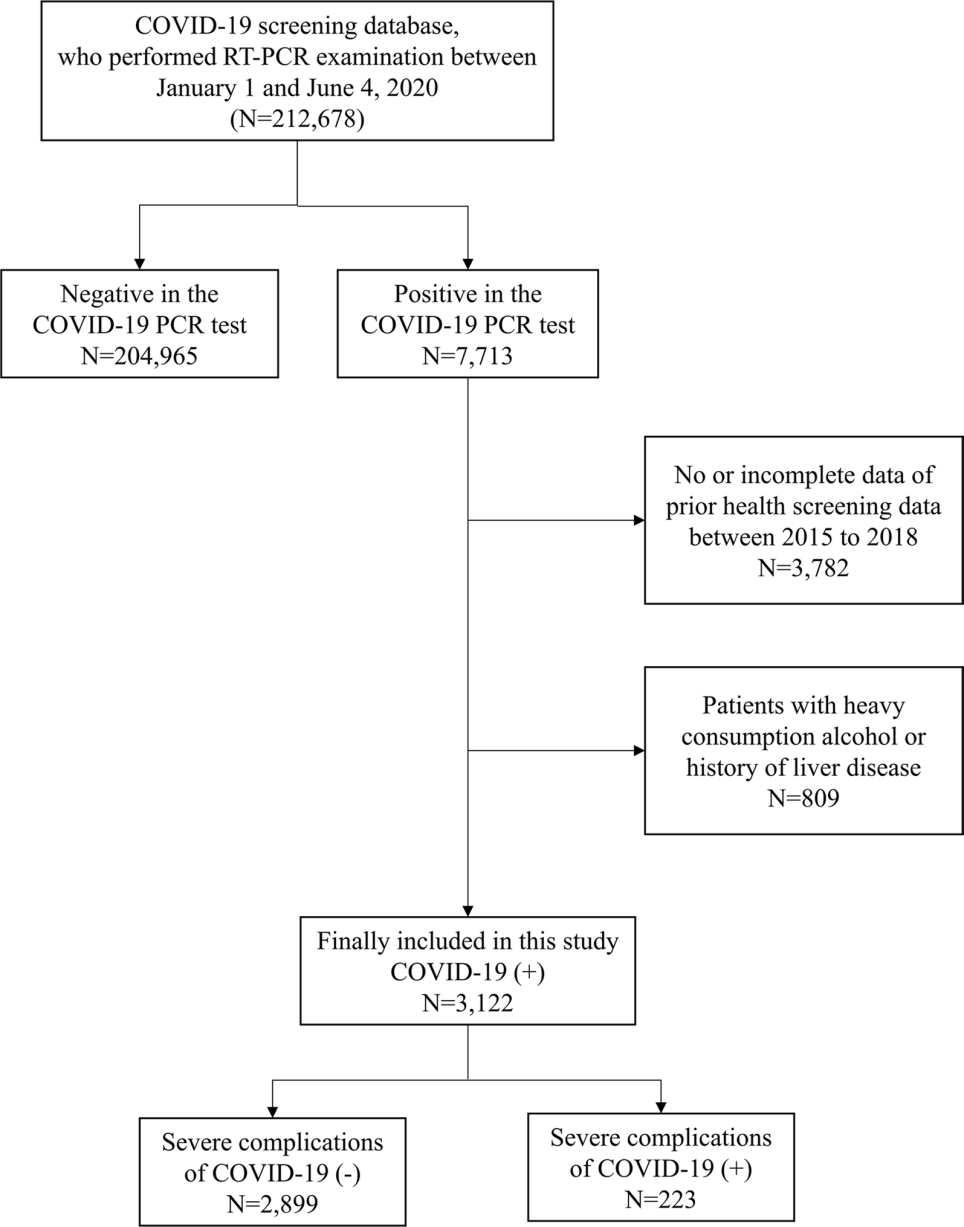
Flow chart depicting patient selection. *COVID-19* coronavirus disease 2019, *RT-PCR* real-time reverse transcription-polymerase chain reaction

In the nationwide COVID-19 cohort dataset from South Korea, there were 212,678 participants aged ≥ 20 who underwent a real-time reverse transcription-polymerase chain reaction (RT-PCR) test for COVID-19. Among them, 7713 patients returned a positive COVID-19 RT-PCR test. From the 7713 patients who suffered a COVID-19 infection, there was data available from the national free health screening program for 3931 of them, from which the FLI was calculated. Patients who either had incomplete or no data for the variables used in the analysis were excluded. Additionally, 809 patients who either conducted heavy alcohol consumption (alcohol drinking ≥ 2 days per week or alcohol intake of > 30 g/day), or had a previous history of liver cirrhosis (International Classification of Diseases 10th Revision (ICD)-10 codes: K70.2, K70.3, K70.4, and K74.6), alcoholic hepatitis (K70.1), chronic viral hepatitis B with delta-agent (B18.0), chronic viral hepatitis B without delta-agent (B18.1), and chronic viral hepatitis C (B18.2), liver and intrahepatic bile duct (C22) were excluded [22, 23]. Finally, 3122 patients were included in this study (Fig. 1).

### Covariate

FLI was calculated by applying the following formula [24].

$${\text{FLI}} = ({\text{e}}^{{0.953 \times {\text{Ln}}\left( {{\text{triglycerides}}} \right) + 0.139 \times {\text{BMI}} + 0.178 \times {\text{Ln}}\left( {{\text{GGT}}} \right) + 0.053 \times {\text{waist}}{\kern 1pt} {\text{circumference}} - 15.745}} )/(1 + {\text{e}}^{{0.953 \times {\text{Ln}}\left( {{\text{triglycerides}}} \right) + 0.139{\text{BMI}} + 0.178 \times {\text{Ln}}\left( {{\text{GGT}}} \right) + 0.053 \times {\text{waist}}{\kern 1pt} {\text{circumference}} - 15.745}} ) \times 100$$

Data on the sex, age, household income level (four quartiles), alcohol consumption, smoking habits, physical activity, BMI, blood pressures, presence of comorbidities, and blood laboratory findings were collected. The data for smoking status (current, former, and never), alcohol consumption (frequency per week), and physical activity (days per week) were acquired. The BMI was expressed as one’s weight (kg) divided by the square of their height (m^2^). Hypertension was outlined when the participants had health claims of blood pressure-lowering agents by diagnostic codes of hypertension (ICD 10: I10–15), blood pressure ≥ 140/90 mmHg, or through a self-report questionnaire regarding hypertension [25]. Diabetes mellitus was defined when participants had either health claims of anti-diabetic drug use with diagnostic codes of diabetes mellitus (ICD 10: E11–14), fasting blood glucose of > 7.0 mmol/L, or positive checking in a self-report questionnaire for diabetes mellitus [26]. Stroke was defined when participants had health claims with ICD-10 of I60–63, I69. Coronary artery disease was defined when participants had health claims with ICD-10 of I20–25 [27]. Dyslipidemia was defined when participants had health claims of lipid-lowing agents with diagnostic codes of dyslipidemia (ICD 10: E78) [28]. Atrial fibrillation was defined when participants had health claims with ICD-10 of I48 [25]. Heart failure was defined when participants had more than 2 health claims with ICD-10 of I10.0, I13.0, I13.2, I25.2, I42.0, I42.9, I43, and I50 [29]. Asthma was defined when participants had more than 2 health claims with an ICD-10 of J45–46 or when participants had health claims of asthma-related agents with ICD-10 codes (J45–46) [30]. Renal disease was defined when participants had health claims with ICD-10 of N17–19, E082, E102, E112, E132, or I12–13 [31]. Malignancy was defined when participants had health claims with an ICD-10 of C00-97 and cancer-specific deductible insurance codes of V027, V193, and V194 [32]. Laboratory findings (aspartate aminotransferase, alanine aminotransferase, GGT, fasting glucose, total cholesterol triglyceride, high-density lipoprotein cholesterol, and low-density lipoprotein cholesterol) were collected from the nationwide health checkups dataset.

### Outcome

The primary outcome was defined as the composite of mechanical ventilation, admission to the intensive care unit (ICU), high-flow oxygen therapy, and mortality during the 2 months after being proven positive for COVID-19. High-flow oxygen therapy was defined as the presence of procedure code with high-flow nasal cannula therapy (M0046). Mechanical ion was either (M5850, M5857, M5858, M5860). The admission to the intensive care unit was described as the presence of the claims code for the intensive care unit (AH110, AH150, AH180-95 AH190-5, AH210, AH250, AH280-9, AH28A, AH290-9, AH380-9, AH38A, AH390-9, AH501, AJ001–AJ011, AJ020-1, AJ031, AJ100–390, AJ2A0, AJ3A0, AJ500–590, V5100, V5200, V5210-20, V5500–5520). Mortality and the timing of death are provided by NHIS and have previously been validated [33, 34].

### Statistical analysis

Since the Asian population is known to have a lower waist circumference and BMI than other races, there is limited data for the optimal cut-off of FLI in the Korean population [35]. Therefore, the statistical analysis was performed by dividing FLI values into tertiles. To evaluate the trend of parameters according to the tertiles of FLT, a Chi-square test was performed to calculate the trend and Jonckheere’s trend test for categorical and continuous variables, respectively.

To investigate the association between FLI levels and the risk of severe complications from COVID-19 infection, univariable and multivariable binary logistic regression were performed. In the multivariable analysis, sex, age, household income level, alcohol consumption, smoking habits, physical activity, BMI, blood pressures, presence of comorbidities, and total cholesterol level were entered as covariates. The results were presented as an odds ratio (OR) and 95% confidence interval (CI). Triglyceride and gamma-glutamyl transferase were already included in the FLI and concerned for multicollinearity among the laboratory parameters, so only alanine aminotransferase and total cholesterol were adjusted.

Secondary outcome analyses were performed by constructing models that had individual severe complications (mechanical ventilation, intensive care unit admission, high-flow oxygen therapy, and death) from COVID-19 as a dependent variable. The subgroup analyses were conducted according to the alcohol intake and BMI (≥ 25 kg/m^2^ vs. < 25 kg/m^2^). In the subgroup analysis, overfitting occurred in the multivariable logistic regression when FLI was entered as tertiles. Consequently, we investigated the OR, 95% CI, and P for interaction relating to an FLI of 60 or higher compared to an FLI less than 60 (as a reference), based on the original classification for non-alcoholic fatty liver disease (NAFLD) with FLI values [36]. To help understand the association between the FLI values and severe complications of COVID-19, we illustrated spline curves representing OR according to the continuous level of FLI values. Statistical analyses were executed using R software, version 3.3.3 (R Foundation for Statistical Computing, Vienna, Austria), and SAS 9.4 version (SAS Inc., Cary, NC, USA). Two-sided *P*-values of less than 0.05 were considered significant.

## Results

### Participants and demographic data

Among the 212,678 participants who underwent COVID-19 testing, this study finally included 3122 COVID-19 patients who had available data for blood laboratory findings. Among the 3122 COVID-19 patients, 959 (30.72%) were male, and 1232 (39.46%) were over 60 years of age. The primary outcome for severe complications of COVID-19 occurred in 223 (7.14%) patients (Table 1). Patients with severe complications were predominantly either the male sex, aged ≥ 60 years, undertook frequent alcohol consumption, exhibited atrial fibrillation, and heart failure. The tertiles of FLI were categorized into T1: 0–< 9.14, T2: 9.14–26.23, and T3: > 26.23. Presence in T3 of FLI was more frequent in patients with severe complications (Table 1). Demographics, comorbidities, and laboratory findings are presented in Table 2. The mean ± standard deviation of FLI was 23.20 ± 21.25. Across the three tertiles of FLI, T3 showed an increased proportion of males, ≥ 60 age, higher house income, former and current smoking history, non-exerciser, higher BMI, and accompanying comorbidities (Table 2).

**Table 1:** Risk factors for severe complications in COVID-19 patients

Variable	Patients without severe complication (N = 2899)	Patients with severe complication (N = 223)	Univariate OR [95% CI]	P-value	Adjusted OR [95% CI]	P-value
Sex, male	838 (28.91)	121 (54.26)	2.92 [2.22–3.84]	< 0.001	1.15 [1.47–3.13]	< 0.001
Age, years						
< 60	1844 (63.61)	46 (20.63)	1 (Ref)	–	1 (Ref)	–
≥ 60	1055 (36.39)	177 (79.37)	6.73 [4.82–9.38]	< 0.001	3.62 [2.49–5.27]	< 0.001
Household income						
Q1, lowest	1064 (36.70)	61 (27.35)	1 (Ref)	–	1 (Ref)	–
Q2	704 (24.28)	45 (20.18)	1.12 [0.75–1.66]	0.591	1.21 [0.79–1.86]	0.376
Q3	491 (16.94)	40 (17.94)	1.42 [0.94–2.15]	0.095	1.35 [0.86–2.11]	0.189
Q4, highest	640 (22.08)	77 (34.53)	2.10 [1.48–2.98]	< 0.001	1.66 [1.13–2.43]	0.010
Alcohol consumption, frequency per week						
None	2310 (79.68)	200 (89.69)	1 (Ref)	–	1 (Ref)	–
About 1 time per week	589 (20.32)	23 (10.31)	0.45 [0.29–0.70]	< 0.001	0.50 [0.31–0.82]	0.006
Smoking habits						
None	2473 (85.31)	152 (68.16)	1 (Ref)	–	1 (Ref)	–
Former smoker	297 (10.24)	59 (26.46)	3.23 [2.34–4.47]	< 0.001	1.68 [1.09–2.59]	0.020
Current smoker	129 (4.45)	12 (5.38)	1.51 [0.82–2.80]	0.186	1.03 [0.52–2.06]	0.928
Physical activity						
< 1 day per week	603 (20.80)	60 (26.90)	1 (Ref)	–	1 (Ref)	–
1–4 days per week	1268 (43.74)	84 (37.67)	0.67 [0.47–0.94]	0.021	0.77 [0.52–1.13]	0.186
≥ 5 days per week	1028 (35.46)	79 (35.43)	0.77 [0.54–1.10]	0.148	0.80 [0.54–1.17]	0.250
Body mass index (kg/m^2^)	23.83 ± 3.33	24.61 ± 3.49	1.07 [1.03–1.11]	< 0.001	–	
Systolic blood pressure, mmHg	120.24 ± 15.09	128.74 ± 14.74	1.04 [1.03–1.04]	< 0.001	–	
Diastolic blood pressure, mmHg	74.22 ± 9.80	78.08 ± 10.03	1.04 [1.03–1.05]	< 0.001	–	
Comorbidities						
Hypertension	882 (30.42)	141 (63.23)	3.93 [2.96–5.22]	< 0.001	1.36 [0.96–1.92]	0.084
Diabetes mellitus	390 (13.45)	73 (32.74)	3.13 [2.32–4.22]	< 0.001	1.40 [0.98–2.00]	0.068
Stroke	99 (3.41)	27 (12.11)	3.90 [2.49–6.11]	< 0.001	1.37 [0.82–2.28]	0.231
Coronary artery disease	132 (4.55)	29 (13.00)	3.13 [2.04–4.81]	< 0.001	1.03 [0.62–1.71]	0.926
Atrial fibrillation	40 (1.38)	21 (9.42)	7.43 [4.30–12.84]	< 0.001	2.21 [1.16–4.21]	0.016
Heart failure	116 (4.00)	43 (19.28)	5.73 [3.92–8.39]	< 0.001	1.85 [1.15–2.96]	0.011
Asthma	157 (5.42)	23 (10.31)	2.01 [1.27–3.18]	< 0.001	1.14 [0.68–1.90]	0.627
Chronic kidney disease	168 (5.80)	33 (14.80)	2.82 [1.89–4.22]	< 0.001	1.21 [0.76–1.92]	0.429
Malignancy	212 (7.31)	32 (14.35)	2.12 [1.42–3.17]	< 0.001	1.37 [0.88–2.14]	0.169
Aspartate aminotransferase, U/L	24.15 ± 11.92	24.97 ± 10.68	1.01 [0.99–1.02]	0.318	–	
Alanine aminotransferase, U/L	22.98 ± 17.94	23.76 ± 14.77	1.00 [0.99–1.01]	0.528	0.99 [0.98–1.00]	0.140
Glutamyl transpeptidase, U/L	25.47 ± 26.44	30.58 ± 26.24	1.01 [1.00–1.02]	0.012	–	–
Fasting glucose, mmol/L	5.53 ± 1.55	6.17 ± 1.77	1.17 [1.09–1.24]	< 0.001	–	–
Total cholesterol, mmol/L	5.06 ± 0.97	4.82 ± 1.04	0.89 [0.83–0.95]	< 0.001	0.95 [0.88–1.02]	0.203
Triglyceride, mmol/L	1.32 ± 0.93	1.58 ± 0.89	1.04 [1.02–1.07]	0.001	–	–
HDL cholesterol, mmol/L	1.47 ± 0.50	1.33 ± 0.35	0.58 [0.48–0.71]	< 0.001	–	–
LDL cholesterol, mmol/L	2.96 ± 0.86	2.76 ± 0.92	0.86 [0.80–0.93]	< 0.001	–	–
Fatty liver index, tertile						
T1	4.74 ± 2.23	5.81 ± 2.28	1 (Ref)	–	1 (Ref)	–
T2	16.52 ± 4.92	17.16 ± 4.48	2.13 [1.40–3.24]	< 0.001	1.24 [0.79–1.97]	0.354
T3	48.05 ± 17.76	49.76 ± 17.50	3.82 [2.58–5.65]	< 0.001	1.77 [1.11–2.82]	0.017

Data are derived from logistic regression analysis for the development of severe complications in COVID-19 patients. Severe complications of COVID-19 is a composite of mechanical ventilation, intensive care unit care, high-flow oxygen therapy, and death. *OR* odds ratio, *CI* confidence interval, *T* tertile, *Q* quartile, *HDL* high-density lipoprotein, *LDL* low-density lipoprotein. Multivariate model is adjusted with sex, age, household income, alcohol consumption, smoking habits, physical activity, body mass index, hypertension, diabetes mellitus, stroke, coronary artery disease, atrial fibrillation, heart failure, asthma, chronic kidney disease, malignancy, alanine aminotransferase, total cholesterol, and tertile of fatty liver index

**Table 2:** Baseline characteristics of COVID-19 patients according to outcome fatty liver index

Variable	Total	Fatty liver index			P-value*
		T1 (< 9.14)	T2 (9.14–26.23)	T3 (> 26.23)	
N	3122	1040	1041	1041	
Fatty liver index, mean	23.20 ± 21.25	4.77 ± 2.24	16.57 ± 4.89	48.24 ± 17.73	< 0.001
Sex, male	959 (30.72)	150 (14.42)	312 (29.97)	497 (47.74)	< 0.001
Age, years					< 0.001
< 60	1890 (60.54)	793 (76.25)	567 (54.47)	530 (50.91)	
≥ 60	1232 (39.46)	247 (23.75)	474 (45.53)	511 (49.09)	
Household income					0.115
Q1, lowest	1125 (36.03)	383 (36.83)	386 (37.08)	356 (34.20)	
Q2	749 (23.99)	273 (26.25)	235 (22.57)	241 (23.15)	
Q3	531 (17.01)	171 (16.44)	178 (17.10)	182 (17.48)	
Q4, highest	717 (22.97)	213 (20.48)	242 (23.25)	262 (25.17)	
Alcohol consumption, frequency per week					0.790
None	2510 (80.40)	838 (80.58)	842 (80.88)	830 (79.73)	
About 1 time per week	612 (19.60)	202 (19.42)	199 (19.12)	211 (20.27)	
Smoking habits					< 0.001
None	2625 (84.08)	967 (92.98)	900 (86.46)	758 (72.81)	
Former smoker	356 (11.40)	54 (5.19)	101 (9.70)	201 (19.31)	
Current smoker	141 (4.52)	19 (1.83)	40 (3.84)	82 (7.88)	
Physical activity					0.001
< 1 day per week	663 (21.24)	185 (17.79)	218 (20.94)	260 (24.98)	
1–4 days per week	1352 (43.31)	479 (46.06)	438 (42.07)	435 (41.79)	
≥ 5 days per week	1107 (35.46)	376 (36.15)	385 (36.98)	346 (33.24)	
Anthropometric measurements					
Body mass index (kg/m^2^)	23.89 ± 3.34	21.08 ± 1.96	23.76 ± 1.96	26.62 ± 3.06	< 0.001
Systolic blood pressure, mmHg	120.85 ± 15.22	113.74 ± 13.66	121.30 ± 14.19	127.49 ± 14.58	< 0.001
Diastolic blood pressure, mmHg	74.49 ± 9.86	70.57 ± 9.12	74.57 ± 9.59	78.33 ± 9.32	< 0.001
Comorbidities					
Hypertension	1023 (32.77)	142 (13.65)	351 (33.72)	530 (50.91)	< 0.001
Diabetes mellitus	463 (14.83)	49 (4.71)	146 (14.02)	268 (25.74)	< 0.001
Stroke	126 (4.04)	22 (2.12)	45 (4.32)	59 (5.67)	< 0.001
Coronary artery disease	161 (5.16)	26 (2.50)	64 (6.15)	71 (6.82)	< 0.001
Atrial fibrillation	61 (1.95)	13 (1.25)	17 (1.63)	31 (2.98)	0.011
Heart failure	159 (5.09)	26 (2.50)	58 (5.57)	75 (7.20)	< 0.001
Asthma	180 (5.77)	41 (3.94)	57 (5.48)	82 (7.88)	< 0.001
Chronic kidney disease	201 (6.44)	32 (3.08)	68 (6.53)	101 (9.70)	< 0.001
Malignancy	244 (7.82)	80 (7.69)	79 (7.59)	85 (8.17)	0.873
Laboratory findings					
Aspartate aminotransferase, U/L	24.21 ± 11.83	21.07 ± 8.61	23.19 ± 8.36	28.35 ± 15.75	< 0.001
Alanine aminotransferase, U/L	23.04 ± 17.73	15.85 ± 9.29	20.93 ± 10.61	32.32 ± 24.54	< 0.001
Glutamyl transpeptidase, U/L	25.84 ± 26.46	15.12 ± 7.01	22.11 ± 12.60	40.27 ± 39.43	< 0.001
Fasting glucose, mg/dL	100.32 ± 28.28	92.94 ± 16.92	99.16 ± 29.03	108.86 ± 33.82	< 0.001
Total cholesterol, mg/dL	194.86 ± 37.60	184.57 ± 32.96	197.79 ± 37.75	202.22 ± 39.56	< 0.001
Triglyceride, mg/dL	118.56 ± 82.09	71.95 ± 27.46	109.52 ± 44.97	174.15 ± 52.65	< 0.001
HDL cholesterol, mg/dL	56.62 ± 18.98	63.38 ± 13.63	56.74 ± 25.42	49.75 ± 12.50	< 0.001
LDL cholesterol, mg/dL	115.00 ± 33.65	106.58 ± 29.79	119.57 ± 33.67	118.93 ± 35.68	< 0.001
Severe complications of COVID-19					
Mechanical ventilation	82 (2.63)	9 (0.87)	26 (2.50)	47 (4.51)	< 0.001
Intensive care unit care	126 (4.04)	19 (1.83)	42 (4.03)	65 (6.24)	< 0.001
High-flow oxygen therapy	75 (2.40)	9 (0.87)	24 (2.31)	42 (4.03)	< 0.001
Death	94 (3.01)	14 (1.35)	25 (2.40)	55 (5.28)	< 0.001
Composite of outcome	223 (7.14)	34 (3.27)	70 (6.72)	119 (11.43)	< 0.001

Data are represented as number of participants (%) or mean ± standard deviation

*T* tertile, *Q* quartile, *HDL* high-density lipoprotein, *LDL* low-density lipoprotein, *TG* triglyceride

^*^P-value is derived from the Jonckheere’s trend test or Chi’s square trend test in patient groups according to tertiles of fatty liver index

### Association of FLI with severe complications of COVID-19

**Fig. 2:**
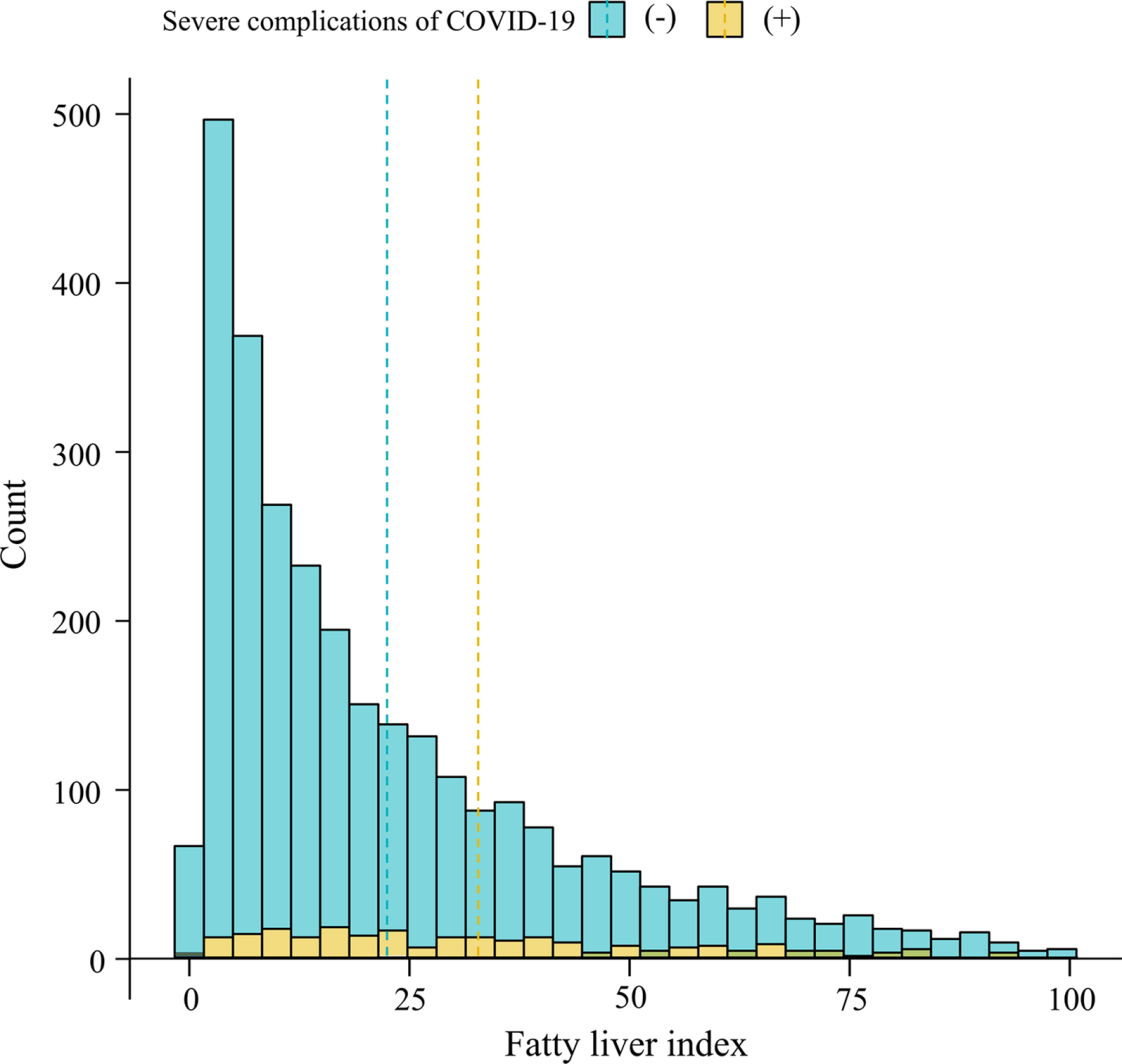
Distribution of fatty liver index according to the presence of severe complications from COVID-19. The vertical dotted lines present the mean values for the fatty liver index in each group with and without severe complications of COVID-19

Among the severe complications of COVID-19, mechanical ventilation was applied to 82 (2.63%) patients, ICU admission in 126 (4.04%) patients, high-flow oxygen therapy in 75 (2.40%) patients, and death in 94 (3.01%) patients, respectively. The distribution of FLI according to the presence of severe complications of COVID-19 was demonstrated in Fig. 2.

In the multivariate logistic analysis, T3 of FLI was positively associated with the risk of severe complications from COVID-19 (adjusted odds ratio (OR): 1.77, 95% confidence interval (CI) (1.11–2.82), P = 0.017) compared to T1 (Table 2). Following the construction of the secondary outcome models regarding individual complications of COVID-19, T3 of FLI was significantly related to mechanical ventilation (adjusted OR: 2.38, 95% CI (1.06–5.35), P = 0.035) and ICU admission (adjusted OR: 1.92, 95% CI (1.06–3.50), P = 0.032) (Table 3). In the subgroup analysis according to alcohol consumption and body mass index, there was no interaction effect on the association of FLI with severe complications of COVID-19 (Table 4).

**Table 3:** Secondary outcome analysis of individual outcomes by fatty liver index

Outcome	Case	Univariate OR [95% CI], T3 versus T1 of FLI	P-value	Adjusted OR [95% CI], T3 versus T1 of FLI	P-value
Mechanical ventilation	82	5.42 [2.64–11.11]	< 0.001	2.38 [1.06–5.35]	0.035
Intensive care unit care	126	3.58 [2.13–6.01]	< 0.001	1.92 [1.06–3.50]	0.032
High-flow oxygen therapy	75	4.82 [2.33–9.95]	< 0.001	1.84 [0.81–4.19]	0.148
Death	94	4.09 [2.26–7.40]	< 0.001	1.43 [0.70–2.92]	0.329

Data are derived from logistic regression analysis for each outcome

Multivariable analysis was adjusted for sex, age, household income, alcohol consumption, smoking habits, physical activity, hypertension, diabetes mellitus, stroke, coronary artery disease, atrial fibrillation, heart failure, asthma, chronic kidney disease, malignancy, alanine aminotransferase, and total cholesterol

Odds ratio (OR) and 95% confidence interval (CI) of each outcome in the third tertile (T3) of fatty liver index (FLI) were compared to the first title (T1) of FLI

**Table 4:** Subgroup analysis according to alcohol consumption and body mass index

Variable	OR [95% CI]^a^	P-value	P for interaction
Alcohol consumption			0.665
No alcohol consumption	2.21 [1.44–3.37]	< 0.001	
Alcohol consumption once per week	1.70 [0.56–5.14]	0.350	
Body mass index (kg/m^2^)			0.271
< 25	3.19 [0.91–11.16]	0.070	
≥ 25	1.51 [0.96–2.38]	0.076	

^a^Data are derived from logistic regression analysis for severe complications of COVID-19

Odds ratio (ORs) and 95% confidence interval (CI) of each severe complications of COVID-19 in patients with fatty liver index 60 or higher comparing with those with fatty liver index less than 60

## Discussion

The key finding of our study is that increased FLI, which represents NAFLD, was associated with a higher risk of severe complications in COVID-19 patients in a general population-based cohort.

In a Chinese hospital-based cohort study with 202 patients, COVID-19 infected patients with NAFLD had a higher risk of disease progression [37]. In a Chinese multicenter retrospective registry study with 66 patients, it was also reported that patients with NAFLD and obesity also presented with severe complications of COVID-19 [38]. In a recent meta-analysis, NAFLD patients had an increased risk of severe COVID-19 infection and admission to the intensive care unit. However, there was no difference in mortality due to COVID-19 among patients with and without NAFLD [39]. Our results correspond with these studies and are important since our key findings demonstrate an association between NAFLD and the development of severe complications from COVID-19 in a large cohort and a general population-based dataset. Conversely, a retrospective study of 155 COVID-19 positive patients in Mexico demonstrated that fatty liver disease and significant liver fibrosis were frequently found in COVID-19 patients but were not independently associated with clinical outcomes [40]. These differences have likely occurred from the differences in the study designs, population type, and ethnicity. Further studies should ultimately be performed to conclude these discrepancies.

Although our study does not investigate the mechanism, some hypotheses may explain the association between FLI and severe COVID-19 outcomes. The receptor of the angiotensin-converting enzyme has a high affinity for the spike protein of COVID-19. Furthermore, the angiotensin-converting enzyme receptor is widely distributed, both in the lung and in hepatobiliary cells. Additionally, it is known that the expression of the angiotensin-converting enzyme receptor increases especially after COVID-19 infection [41]. Therefore, hyperactivation of the receptor of the angiotensin-converting enzyme-mediated immune system elicited by pulmonary infarction can cause collateral damage to hepatocytes [42, 43]. Although data was not collected on insulin resistance, for example, the HOMA index, NAFLD is closely related to insulin resistance. Since insulin resistance is related to various comorbidities and the inflammatory cascade associated with the cytokine storm, hyper-activation of the systemic inflammatory reaction caused by COVID-19 infection may be triggered in insulin resistance situations, which may lead to serious complications [44].

Our study has limitations. Firstly, the retrospective cohort design of this study led to an inability to prove the causal relationship. Secondly, in the health screening cohort, young people under the age of 40 could not be included, which led to the introduction of selection bias. Thirdly, it is difficult to generalize our results for overall ethnicity because our dataset consists of only the Korean general population. Fourth, our dataset checked the lipid profile before COVID-19 infection, therefore, the serial change of the lipid profile following COVID-19 infection could not be confirmed. Fifth, the body mass index and waist circumference, which are major components of FLI were retrospectively acquired. Moreover, the time gap between body measurements in health screening and COVID-19 infection may cause the body mass index and waist circumference to fluctuate. Sixth, the various times of acquisition for the information/data from which FLI was calculated before COVID-19 infection represents another limitation. Finally, our dataset did not directly confirm NAFLD through biopsy or ultrasound.

## Conclusions

Our study demonstrated that FLI, which represents NAFLD, was positively associated with an increased risk of severe complications of COVID-19. FLI might be used as a prognostic marker for the severity of COVID-19.

## Data Availability

The COVID-19 dataset supporting the conclusions of this article is available on the homepage of the National Health Insurance Sharing Service [http://nhiss.nhis.or.kr/bd/ab/bdaba021eng.do]. To gain access to the data, a completed application form, a research proposal, and the applicant’s approval document from the institutional review board should be submitted to and reviewed by the inquiry committee of research support in NHIS. Currently, the use of NHIS data is allowed only for Korean researchers.

## Abbreviations

COVID-19: Coronavirus disease 2019
SARS-CoV-2: Severe acute respiratory syndrome coronavirus 2
NAFLD: Non-alcoholic fatty liver disease
FLI: Fatty liver index
BMI: Body mass index
GGT: Gamma-glutamyl transferase
RT-PCR: Reverse transcription-polymerase chain reaction
ICD: International Classification of Diseases
ICU: Intensive care unit
OR: Odds ratio
CI: Confidence interval

## References

[1] Reid M, Abdool-Karim Q, Geng E, Goosby E (2021). How will COVID-19 transform global health post-pandemic? Defining research and investment opportunities and priorities. PLoS Med.

[2] Wiersinga WJ, Rhodes A, Cheng AC, Peacock SJ, Prescott HC (2020). Pathophysiology, transmission, diagnosis, and treatment of coronavirus disease 2019 (COVID-19): a review. JAMA.

[3] WHO Coronavirus (COVID-19) Dashboard. https://covid19.who.int.

[4] Chen T, Wu D, Chen H, Yan W, Yang D, Chen G (2020). Clinical characteristics of 113 deceased patients with coronavirus disease 2019: retrospective study. BMJ.

[5] Jordan RE, Adab P, Cheng KK (2020). Covid-19: risk factors for severe disease and death. BMJ.

[6] Pakhchanian H, Raiker R, Mukherjee A, Khan A, Singh S, Chatterjee A (2021). Outcomes of COVID-19 in CKD patients. A multicenter electronic medical record cohort study. Clin J Am Soc Nephrol.

[7] Clark A, Jit M, Warren-Gash C, Guthrie B, Wang HHX, Mercer SW (2020). Global, regional, and national estimates of the population at increased risk of severe COVID-19 due to underlying health conditions in 2020: a modelling study. Lancet Glob Health.

[8] Metrakos P, Nilsson T (2018). Non-alcoholic fatty liver disease—a chronic disease of the 21st century. J Biomed Res.

[9] Lonardo A, Sookoian S, Pirola CJ, Targher G (2016). Non-alcoholic fatty liver disease and risk of cardiovascular disease. Metabolism.

[10] Huh JH, Ahn SV, Koh SB, Choi E, Kim JY, Sung KC (2015). A prospective study of fatty liver index and incident hypertension: the KoGES-ARIRANG study. PLoS ONE.

[11] Yadav D, Choi E, Ahn SV, Koh SB, Sung KC, Kim JY (2016). Fatty liver index as a simple predictor of incident diabetes from the KoGES-ARIRANG study. Medicine (Baltimore).

[12] Huh JH, Kim JY, Choi E, Kim JS, Chang Y, Sung KC (2017). The fatty liver index as a predictor of incident chronic kidney disease in a 10-year prospective cohort study. PLoS ONE.

[13] Bedogni G, Bellentani S, Miglioli L, Masutti F, Passalacqua M, Castiglione A (2006). The Fatty Liver Index: a simple and accurate predictor of hepatic steatosis in the general population. BMC Gastroenterol.

[14] Sviklāne L, Olmane E, Dzērve Z, Kupčs K, Pīrāgs V, Sokolovska J (2018). Fatty liver index and hepatic steatosis index for prediction of non-alcoholic fatty liver disease in type 1 diabetes. J Gastroenterol Hepatol.

[15] Kahl S, Straßburger K, Nowotny B, Livingstone R, Klüppelholz B, Keßel K (2014). Comparison of liver fat indices for the diagnosis of hepatic steatosis and insulin resistance. PLoS ONE.

[16] Lee SW, Ha EK, Yeniova A, Moon SY, Kim SY, Koh HY (2021). Severe clinical outcomes of COVID-19 associated with proton pump inhibitors: a nationwide cohort study with propensity score matching. Gut.

[17] Lee SW, Yang JM, Moon SY, Yoo IK, Ha EK, Kim SY (2020). Association between mental illness and COVID-19 susceptibility and clinical outcomes in South Korea: a nationwide cohort study. Lancet Psychiatry.

[18] Seong SC, Kim YY, Park SK, Khang YH, Kim HC, Park JH (2017). Cohort profile: the National Health Insurance Service-National Health Screening Cohort (NHIS-HEALS) in Korea. BMJ Open.

[19] Song TJ, Kim JW, Kim J (2020). Oral health and changes in lipid profile: a nationwide cohort study. J Clin Periodontol.

[20] Chang Y, Woo HG, Lee JS, Song TJ (2021). Better oral hygiene is associated with lower risk of stroke. J Periodontol.

[21] Chang Y, Lee JS, Lee KJ, Woo HG, Song TJ (2020). Improved oral hygiene is associated with decreased risk of new-onset diabetes: a nationwide population-based cohort study. Diabetologia.

[22] Lee CH, Han KD, Kim DH, Kwak MS (2021). The repeatedly elevated fatty liver index is associated with increased mortality: a population-based cohort study. Front Endocrinol (Lausanne).

[23] Kim JH, Moon JS, Byun SJ, Lee JH, Kang DR, Sung KC (2020). Fatty liver index and development of cardiovascular disease in Koreans without pre-existing myocardial infarction and ischemic stroke: a large population-based study. Cardiovasc Diabetol.

[24] Koehler EM, Schouten JN, Hansen BE, Hofman A, Stricker BH, Janssen HL (2013). External validation of the fatty liver index for identifying nonalcoholic fatty liver disease in a population-based study. Clin Gastroenterol Hepatol.

[25] Woo M-H, Lee HS, Kim J (2019). Effect of pioglitazone in acute ischemic stroke patients with diabetes mellitus: a nested case-control study. Cardiovasc Diabetol.

[26] Kim J, Hyun HJ, Choi EA, Kim Y, Bae YJ, Kang HT (2020). Metformin use reduced the risk of stomach cancer in diabetic patients in Korea: an analysis of Korean NHIS-HEALS database. Gastric Cancer.

[27] Oh TK, Song IA (2021). Association between the weekday on which coronary artery bypass graft surgery is performed and the 30-day mortality: a nationwide cohort study in South Korea. J Card Surg.

[28] Seo MH, Kim Y-H, Han K, Jung J-H, Park Y-G, Lee S-S (2018). Prevalence of obesity and incidence of obesity-related comorbidities in Koreans based on National Health Insurance Service Health checkup data 2006–2015. J Obes Metab Syndr.

[29] Roh JH, Park JH, Lee H, Yoon YH, Kim M, Kim YG (2020). Higher fatty liver index is associated with increased risk of new onset heart failure in healthy adults: a nationwide population-based study in Korea. BMC Cardiovasc Disord.

[30] Kim JH, Wee JH, Choi HG, Park JY, Hwang YI, Jang SH (2021). Association between statin medication and asthma/asthma exacerbation in a national health screening cohort. J Allergy Clin Immunol Pract.

[31] Kim KM, Oh HJ, Choi HY, Lee H, Ryu DR (2019). Impact of chronic kidney disease on mortality: a nationwide cohort study. Kidney Res Clin Pract.

[32] Lim H, Lee YH, Bae S, Koh DH, Yoon M, Lee BE (2021). Cancer cluster among small village residents near the fertilizer plant in Korea. PLoS ONE.

[33] Song TJ, Jeon J, Kim J (2021). Cardiovascular risks of periodontitis and oral hygiene indicators in patients with diabetes mellitus. Diabetes Metab.

[34] Lee J, Lee JS, Park S-H, Shin SA, Kim K (2016). Cohort Profile: The National Health Insurance Service-National Sample Cohort (NHIS-NSC), South Korea. Int J Epidemiol.

[35] Grundy SM, Cleeman JI, Daniels SR, Donato KA, Eckel RH, Franklin BA (2005). Diagnosis and management of the metabolic syndrome: an American Heart Association/National Heart, Lung, and Blood Institute Scientific Statement. Circulation.

[36] Wong VW, Wong GL, Chan RS, Shu SS, Cheung BH, Li LS (2018). Beneficial effects of lifestyle intervention in non-obese patients with non-alcoholic fatty liver disease. J Hepatol.

[37] Ji D, Qin E, Xu J, Zhang D, Cheng G, Wang Y (2020). Non-alcoholic fatty liver diseases in patients with COVID-19: a retrospective study. J Hepatol.

[38] Zheng KI, Gao F, Wang X-B, Sun Q-F, Pan K-H, Wang T-Y (2020). Letter to the editor: Obesity as a risk factor for greater severity of COVID-19 in patients with metabolic associated fatty liver disease. Metab Clin Exp.

[39] Singh A, Hussain S, Antony B (2021). Non-alcoholic fatty liver disease and clinical outcomes in patients with COVID-19: a comprehensive systematic review and meta-analysis. Diabetes Metab Syndr.

[40] Lopez-Mendez I, Aquino-Matus J, Gall SM-B, Prieto-Nava JD, Juarez-Hernandez E, Uribe M (2021). Association of liver steatosis and fibrosis with clinical outcomes in patients with SARS-CoV-2 infection (COVID-19). Ann Hepatol.

[41] Zou X, Chen K, Zou J, Han P, Hao J, Han Z (2020). Single-cell RNA-seq data analysis on the receptor ACE2 expression reveals the potential risk of different human organs vulnerable to 2019-nCoV infection. Front Med.

[42] Xu Z, Shi L, Wang Y, Zhang J, Huang L, Zhang C (2020). Pathological findings of COVID-19 associated with acute respiratory distress syndrome. Lancet Respir Med.

[43] Polakos NK, Cornejo JC, Murray DA, Wright KO, Treanor JJ, Crispe IN (2006). Kupffer cell-dependent hepatitis occurs during influenza infection. Am J Pathol.

[44] de Luca C, Olefsky JM (2008). Inflammation and insulin resistance. FEBS Lett.

